# Estimation of Greenhouse Gas Emission Factor During Hanwoo Manure Composting

**DOI:** 10.3390/ani16203322

**Published:** 2026-10-21

**Authors:** Woo Je Lee, Geun Woo Park, Sung Chul Seo, Ki-Youn Kim, Jung Kon Kim

**Affiliations:** 1 Department of Safety Engineering, Seoul National University of Science and Technology, Seoul 01811, Republic of Korea; 34uj@seoultech.ac.kr; 2 Department of Animal Industry Convergence, Kangwon National University, Chuncheon 25913, Republic of Korea; rmsdn92@kangwon.ac.kr; 3 Center for Health Related Aerosol Studies, University of Cincinnati, Cincinnati, OH 45221, USA; scseo@uc.edu; 4 Smart Animal Environment Division, National Institute of Animal Science, Rural Development Administration, Wanju 54875, Republic of Korea

**Keywords:** methane, nitrous oxide, flux chamber, seasonal variation

## Abstract

Accurate estimation of climate-altering gases, such as methane and nitrous oxide, from livestock waste treatment is critical for establishing national inventories. This study aims to monitor and analyze the emission characteristics of these gases during the treatment process at an operational Hanwoo (Korean cattle for beef) farm in Republic of Korea to develop country-specific metrics. A full-scale compost shed was modified into a 2400 m^3^ flux chamber equipped with fans to control airflow at 183 m^3^·min^−1^. Real-time gas concentrations were measured using enhanced-performance laser-based analyzers across summer, autumn, and winter. The results indicated that mean emissions for both methane and nitrous oxide were highest in summer, followed by winter, and lowest in autumn. Daily patterns revealed higher hourly mean emissions during the morning and afternoon. The final estimated emission factors were 1.28 kgCH_4_·head^−1^·yr^−1^ and 0.039 kgN_2_O· head^−1^·yr^−1^. These values are relatively lower than those reported in previous studies. These values were lower than those reported in several previous studies. Differences in facility scale and operating conditions may partly explain this result; however, potential underestimation due to non-uniform airflow should also be considered.

## Simple Summary

Raising farm animals produces a large amount of greenhouse gases, mostly from the animals themselves and their manure, which contributes to global climate change. However, the exact amount of gas released can change greatly depending on the type of animal and how it is raised in different countries. The problem is that current international standards may not accurately reflect these local differences. In this study, our goal was to measure exactly how much greenhouse gas comes from the manure of Hanwoo, which is Korean native cattle. We found that the amount of gas produced by Hanwoo manure is actually much lower than what current international standards suggest. This means that using global averages overestimates the environmental impact of Hanwoo farming. By using our specific measurements, Korea can update its environmental guidelines for managing Hanwoo manure. This will allow society to predict greenhouse gas levels much more accurately and make better decisions for protecting the environment.

## 1. Introduction

The growth of agriculture and livestock industries, along with rising animal populations, is a major source of greenhouse gases like methane (CH_4_) and nitrous oxide (N_2_O) [1,2]. In particular, livestock manure treatment processes like storage and composting are critical hotspots generating methane and nitrous oxide [3,4,5].

Greenhouse gases released into the atmosphere accelerate global warming. Therefore, accurately estimating emissions and evaluating environmental impacts in the livestock sector have become essential tasks [6]. Globally, researchers widely use default emission factors from the Intergovernmental Panel on Climate Change (IPCC) and integrated global databases like DATAMAN to estimate greenhouse gas emissions [7,8].

However, relying on generic default factors introduces substantial uncertainty. Meta-analyses and field studies show that gas emission characteristics vary significantly depending on the rearing environment, housing system, feed quality, and manure management method [9,10,11].

For example, dairy farm studies in Denmark and the United Kingdom confirmed that current guidelines and national inventory models significantly underestimate methane emissions from manure storage facilities [12,13]. Field measurements in Kenya and South Africa showed distinct differences from IPCC default values due to local climates and specific feeding constraints, such as seasonal feed scarcity, feeding below maintenance, and low-quality tropical forages. Consequently, South African enteric methane EFs for dairy (71.8–76.4 kg/head/year) and beef cattle (62.4–78.9 kg/head/year) were much higher than the IPCC defaults (46 and 31 kg/head/year) [14]. In contrast, Kenyan manure N_2_O EFs (0.38–0.44%) and CH_4_ EFs were significantly lower than the IPCC defaults (1.0% and 4.4 g/kg VS, respectively) [15]. Secondary evidence from other livestock systems—such as Australian broiler and swine facilities, and UK pasture soils—similarly highlights the limitations of relying on default emission factors [16,17,18]. Just as regional characteristics and measurement methods cause significant variations in these cases, locally derived emission factors are equally essential for cattle manure composting in Korea.

In Republic of Korea, economic growth and rising meat consumption have caused a sharp increase in livestock numbers and manure production [19]. To achieve carbon neutrality by 2050, Republic of Korea is developing country-specific activity data and emission factors [20]. These factors will enhance the accuracy of national inventories, enable reliable emission quantification, and support effective mitigation strategies in the livestock sector. Some baseline studies have analyzed non-CO_2_ greenhouse gas emission trends, developed life-cycle emission factors for swine manure, and established livestock inventories [21,22,23]. However, existing studies and available data often fail to adequately account for real-field variables. Consequently, country-specific data derived from full-scale operational facilities remain insufficient to further refine and improve the accuracy of the current national inventory.

As of 2021, the livestock sector represents a high proportion of Republic of Korea’s agricultural production value. It includes five of the top ten agricultural commodities by production value: pigs, Hanwoo and beef cattle, eggs, chickens, and milk [24]. According to Korean livestock manure treatment statistics, approximately 83.8% of the total generated manure of all livestock is treated through composting [25]. Thus, estimating greenhouse gas emissions from composting facilities is vital for building the national greenhouse gas inventory.

However, long-term field data measuring greenhouse gas emissions directly from Korean livestock housing or composting facilities are limited. Seunggun Won et al. measured greenhouse gas emissions from Hanwoo manure using flux chambers, but their study was limited to a single measurement [26]. Although emission factor studies using small-scale chambers exist, they failed to account for field variables such as agitation [27]. Technical limitations have also hindered accurate measurements, such as gas dispersion outside the facilities [28]. Consequently, there is a growing need to evaluate emissions from individual farm-scale treatment facilities and establish national emission factors using precise chamber methods or dispersion models.

Therefore, this study monitors the manure treatment process of an operational domestic livestock farm to clearly analyze greenhouse gas (methane and nitrous oxide) emission characteristics. Currently, in accordance with the 2006 IPCC Guidelines, Republic of Korea utilizes emission factors for Hanwoo manure management as 1 kgCH_4_·head^−1^·year^−1^ for CH_4_ and 50.13 kgN·head^−1^·year^−1^ for N_2_O. As these correspond to the Tier 1 level, we aim to apply Tier 2 emission factors that reflect the specific variables of Korean Hanwoo farms.

Based on our results, we aim to estimate country-specific emission factors suited to the Korean environment. Ultimately, this research will contribute to establishing air quality improvement policies and emission reduction strategies for the livestock sector.

## 2. Materials and Methods

**Figure 1:**
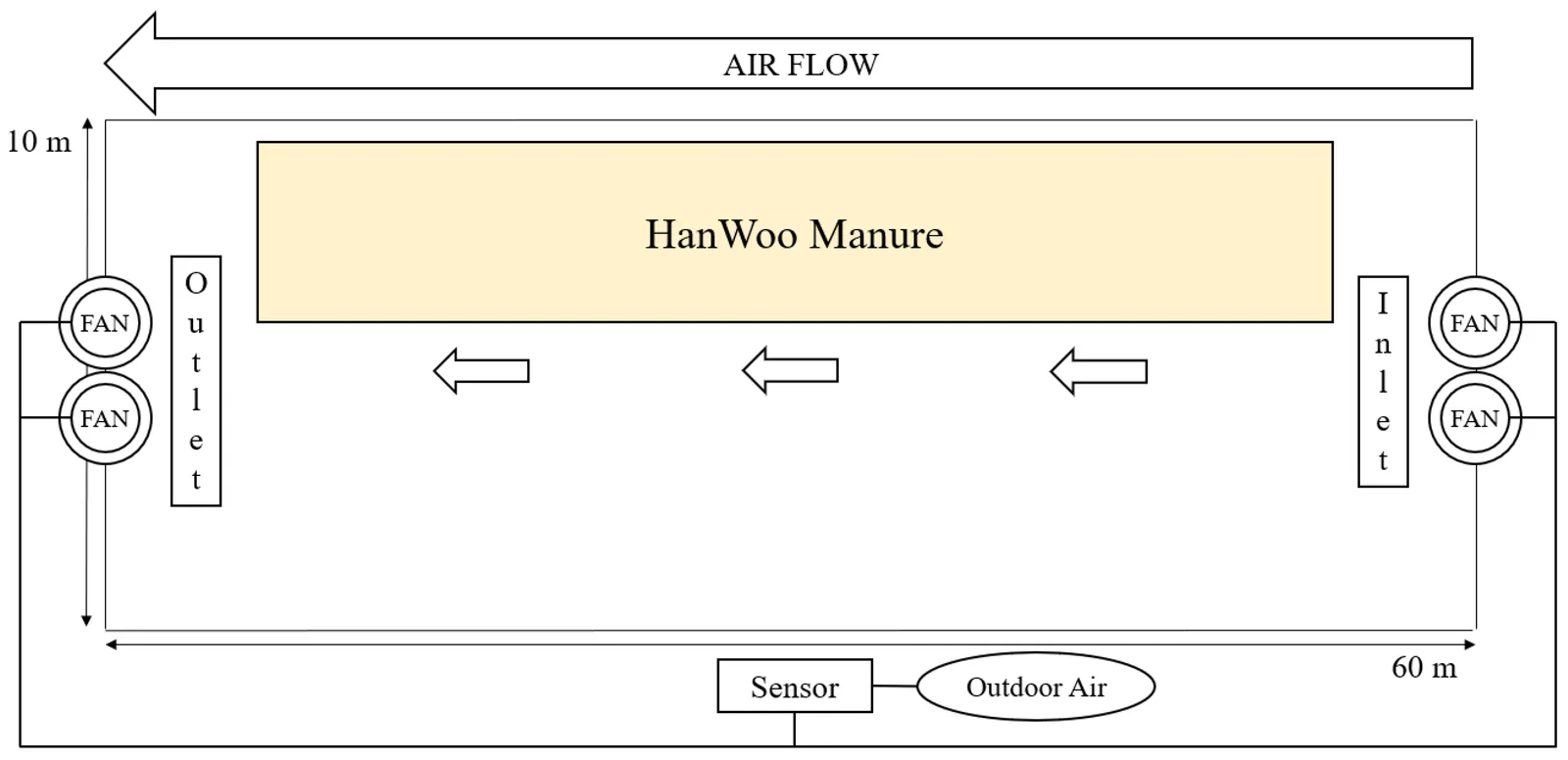
Hanwoo manure flux chamber. The arrows indicate the direction of airflow from the inlet to the outlet.

**Figure 2:**
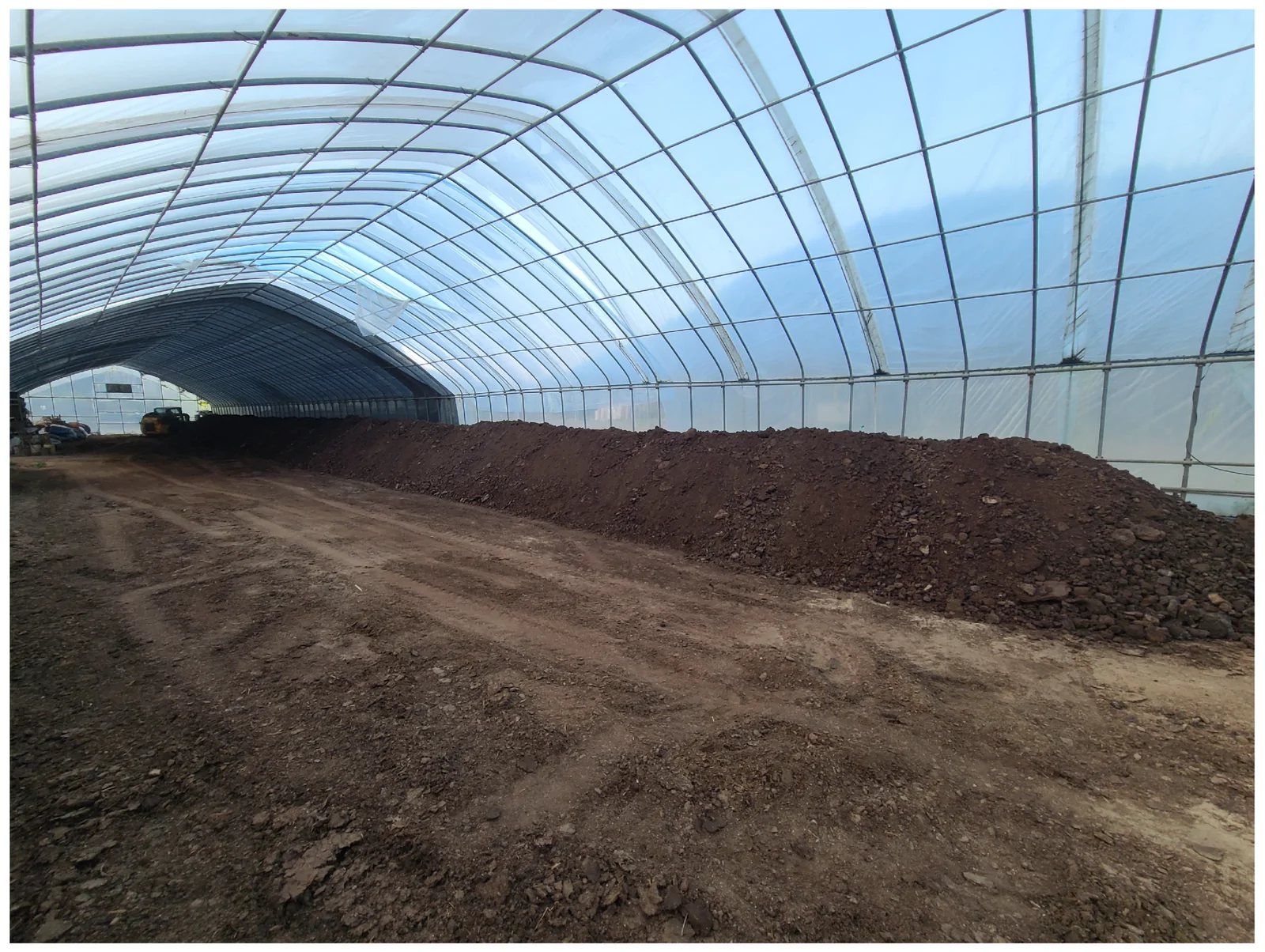
Picture of flux chamber.

The overall research methodology followed the same procedure described in our previous study [29]. To measure greenhouse gas emissions, we modified a compost shed at a Hanwoo cattle farm in Chuncheon, Gangwon-do, Republic of Korea, into a flux chamber (Figure 1 and Figure 2). We controlled the chamber’s airflow by installing two fans at the inlet and two fans at the outlet. Each fan measured 62 by 62 cm with an airflow capacity of 183 m^3^·min^−1^.

Areas around the installed fans, all openings, and tears were sealed completely using plastic sheets and tape. For doors, air leakage was prevented using plastic tape when there was no entry or exit. During entry and exit, the doors were opened and closed as quickly as possible, and internal tasks (e.g., checking compost, turning) were performed after resealing with tape. The flux chamber had a width of 10 m, a length of 60 m, and an average height of 4 m, yielding a total volume of 2400 m^3^. For the composting process, the manure pile was stacked on one side of the flux chamber, measuring about 2 m wide, 40 m long, and 2 m high. However, due to the size of the chamber, an airtightness test was not conducted. As a result, uncontrolled air exchange may have affected the estimation of emissions.

Following the farm’s standard operating procedures, a tractor-mounted loader turned the compost once every 2 to 3 days. This process involves digging out the bottom of the compost pile to flip and mix the upper and lower layers. The mixed compost was reshaped into its original form to complete the turning work. This entire process took approximately 30 min.

Previous studies using flux chambers typically set the air changes per hour (ACH) between 10 and 60 [30,31]. Therefore, we designed this experiment to maintain a similar range. For this purpose, we utilized fans (Shinan Greentech Co., Ltd., Suncheon, Republic of Korea) with a size of 62 cm × 62 cm and an airflow capacity of 183 m^3^·min^−1^. Considering the available electrical power at the test farm, we set the maximum fan capacity, which achieved an ACH of 9.15.

To monitor greenhouse gases, we used enhanced performance QC rack-mounted gas analyzers (GLA351-N2OM1, ABB, Zürich, Switzerland) based on a laser method. Real-time measurements were conducted by connecting the sensor to five points: two inlet fans, two outlet fans, and one location outside the chamber. Measurements were taken for 10 min per section across all five sections. To compensate for the inability to measure the inlet and outlet simultaneously, the measurement sequence was conducted as follows: inlet point 1—outlet point 1—inlet point 2—outlet point 2—outside. One complete measurement cycle required approximately 50 min. For each cycle, the mean concentration measured at the two outlet points and the mean concentration measured at the two inlet points were used to calculate the outlet–inlet concentration difference. The sensor was calibrated before and after the entire measurement period. Due to the extended measurement duration in winter, we performed six calibrations in total, once every 10 days.

After measuring gas concentrations at the inlet and outlet of the flux chamber, we subtracted the inlet concentration from the outlet concentration. This calculation determined the net gas concentration generated during composting. Next, we calculated the gas emission rate µg·m^−3^ using the formula: (gas concentration × atmospheric pressure × molecular weight)/(air temperature × gas constant). Finally, we applied the chamber airflow rate and the weight of the Hanwoo manure to the emission rate. This estimated the final emission factor (µg·s^−1^·ton^−1^), representing the gas emitted per second per ton of Hanwoo manure.

Atmospheric pressure data for the experimental dates were collected from the Korea Meteorological Administration. The air temperature around the flux chamber was recorded using a thermometer. The detailed mathematical equation for estimating the emission factor is as follows:

(1) $$\mathrm{EF}=\;\frac{(C_{\mathrm{out}}-C_{\mathrm{in}})\times 10^{-9}\times \mathrm{atm}\times \mathrm{MW}}{T\times R}\times Q÷W\times 10^{6}$$

EF: Emission Factor (µg·s^−1^·ton^−1^);

C_out_: Gas Concentration of Outlet (ppb);

C_in_: Gas Concentration of Inlet (ppb);

Atm: Atmospheric pressure (Pa);

MW: Molecular weight of gases (CH_4_: 16.04 g·mol^−1^, N_2_O: 44.01 g·mol^−1^);

T: Temperature (K);

R: Gas Constant (8.314 J/(mol·K));

Q: Flow rate of fan (6.1 m^3^·s^−1^);

W: Weight of Hanwoo manure (ton).

Although we aimed to measure emissions across all four seasons in Republic of Korea, we selected autumn to represent both spring and autumn due to their climatic similarities. Previous field studies in Republic of Korea provide indirect support for similarities in gas concentrations between spring and autumn. Kim et al. reported no statistically significant differences in ammonia and hydrogen sulfide concentrations between these seasons in mechanically ventilated swine houses (*p* > 0.05) [32]. Similarly, Oh et al. reported mean atmospheric ammonia concentrations of 21.7 ± 11.8 ppb in spring and 22.2 ± 13.6 ppb in autumn, averaged across two rural monitoring sites in Jeongeup [33]. These findings provide contextual support for considering autumn as a proxy for spring. In our study, the average spring temperature and humidity in Chuncheon were 13.2 °C and 59%, respectively. These values were highly similar to the autumn ambient temperature of 13.6 °C and humidity of 50% around the chamber. However, similar ambient conditions do not establish comparable CH_4_ or N_2_O emissions during manure composting. Accordingly, autumn was not regarded as a validated substitute for spring emission measurements. Therefore, in this study, autumn was used as a rough proxy for climate rather than a complete substitute for spring.

The measurement periods for each season were September 2024 for summer (9 days total), October 2024 for autumn (7 days total), and January to March 2025 for winter (67 days total). Based on the most recent 30-year climate data, the Korea Meteorological Administration reported September 26 as the average onset date of autumn in Republic of Korea [34]. Therefore, early September measurements were classified as summer. Most of the winter measurement period occurred during winter, although the final portion extended into early spring because monitoring continued until the compost reached full maturity.

For the composting process, Hanwoo manure was mixed with sawdust. In accordance with standard practices of Korean Hanwoo farms, sawdust was mixed to account for 25–30% of the total compost weight before being loaded into the flux chamber. The total weight was 111 tons in summer and autumn (83 tons of manure and 28 tons of sawdust). In winter, the total weight was 204 tons (143 tons of manure and 61 tons of sawdust). Each monitoring period continued from the initial loading into the compost shed until the predefined chemical criteria for terminating monitoring were met.

This study comprised three composting batches at the same farm, with one batch monitored in each of summer, autumn, and winter. Each batch involved a single manure loading, without subsequent manure addition or replacement, and constituted the experimental unit. Successive emission-factor estimates were repeated observations within the same batch rather than independent experimental replicates. Thus, there was no independent batch replication within any season.

The C/N ratio [35,36] and the volatile solids (VS) reduction rate [37] were used as operational criteria for determining the end of each monitoring period. Monitoring was terminated when the C/N ratio dropped below 20 and the VS reduction rate reached at least 50% compared to the initial state. The entire compost pile was divided into five zones, and a 200 g sample was collected from each zone. Sampling was conducted in the morning every day during summer and autumn, and once every three days in winter. The collected samples were transported to the laboratory for analysis. The average analytical values from the five zones were used as the overall C/N ratio and VS content of the compost pile.

Statistical analyses were performed using Python (ver. 3.14) and its related libraries (pandas, NumPy, and SciPy). We used the Shapiro–Wilk test and Levene’s test to evaluate data normality and homogeneity of variance. Negative emission-factor values were excluded. Upper outliers were identified separately for each gas and season and were defined as observations exceeding (Q3 + 1.5IQR). Because the data violated both assumptions, we applied the non-parametric Kruskal–Wallis test to determine significance between groups. Dunn’s test with Holm adjustment for multiple comparisons was used for post hoc testing, and the statistical significance level for all tests was set at *p* < 0.05.

Within-batch temporal dependence was evaluated separately for each gas and composting batch. Emission-factor values were natural-log-transformed, and temporal trends were represented using a natural cubic spline of elapsed composting time with four basis functions. Residual dependence was modeled using a continuous-time exponential correlation structure based on the original observation timestamps. Models were estimated by maximum likelihood using SciPy and compared with temporally independent models using AIC. Remaining dependence was evaluated from correlations between adjacent standardized one-step prediction errors.

## 3. Results and Discussion

### 3.1. General Measurement Data

Table 1 shows the moisture content, total Kjeldahl nitrogen (TKN), volatile solids, C/N ratio, and pH for each season. At the end of each monitoring period, the C/N ratio was below 20, and the VS content had decreased by at least 50% relative to its initial value. Thus, all batches met the predefined chemical criteria for terminating monitoring.

The ambient temperature conditions during each monitoring period are summarized in Table 2. The temperature ranged from 19.0 to 31.5 °C during summer, with a mean of 24.2 °C; from 7.5 to 22.4 °C during autumn, with a mean of 13.6 °C; and from −17.9 to 15.0 °C during winter, with a mean of −1.5 °C.

### 3.2. Measurement of Greenhouse Gas Emissions by Season

Table 3 summarizes the greenhouse gas emissions measured in different seasons. For both methane and nitrous oxide, the mean emissions were highest in summer, followed by winter, and lowest in autumn. The Kruskal–Wallis test revealed statistically significant differences in CH_4_ emissions among the three seasons at the *p* < 0.001 level. Dunn’s tests with Holm adjustment identified nominal differences in all three pairwise comparisons (all adjusted *p* < 0.001).

For N_2_O, significant differences were observed between three seasons (*p* = 0.017). However, Dunn’s tests with Holm adjustment identified a nominal difference only between the summer and autumn (*p* = 0.018). The summer–winter (*p* = 0.057) and autumn–winter (*p* = 0.414) comparisons were not nominally significant. Autumn and winter were classified into the same statistical group despite showing differences in their means and medians. This outcome stems from the nature of the Kruskal–Wallis test and its post hoc procedures. Because this method analyzes rank differences, it can yield results that differ from visual differences in means or medians [38,39].

The unequal numbers of retained observations resulted in a greater contribution from the winter batch to the pooled means: 45.79% for CH_4_ and 43.79% for N_2_O. These pooled means summarize the available observations under the monitored conditions. However, the observation-based weights do not necessarily reflect the annual distribution of manure throughput or composting activity. Consequently, annual estimates derived from these pooled means should be interpreted as preliminary extrapolations from the monitored batches, with uncertainty arising from unequal temporal coverage and the absence of activity-based seasonal weighting.

Substantial within-batch temporal dependence was detected for both gases. Before accounting for residual temporal correlation, correlations between adjacent trend-adjusted residuals ranged from 0.324 to 0.765 for CH_4_ and from 0.590 to 0.955 for N_2_O. Compared with models assuming temporally independent residuals, the continuous-time exponential correlation models produced substantially lower AIC values for all gas–batch combinations (ΔAIC = −943.4 to −60.3).

The estimated correlations between observations separated by 50 min ranged from 0.559 to 0.777 for CH_4_ and from 0.616 to 0.956 for N_2_O. The strongest temporal persistence was found for N_2_O during the winter batch, with a correlation of 0.956 and a correlation decay time of approximately 50.4 h. After accounting for the continuous-time correlation structure, correlations between adjacent standardized one-step prediction errors ranged from −0.168 to 0.029, indicating that most of the positive short-term temporal dependence had been accounted for by the models.

The temporal-correlation analysis showed that successive emission-factor estimates within each composting batch were not statistically independent. Consequently, the effective amount of independent information was smaller than suggested by the total number of observations. The nominal *p*-values from the Kruskal–Wallis and Dunn tests may therefore overstate the certainty of the differences if every measurement is interpreted as an independent replicate.

In addition, only one composting batch was monitored during each season. Seasonal conditions and batch-specific characteristics were therefore completely confounded, and between-batch variability within a season could not be estimated. Accordingly, the rank-based results should be interpreted as exploratory evidence of differences among the three monitored composting batches and not as definitive evidence of general seasonal effects. The consistently positive temporal correlation obtained using different gap definitions and temporal-trend specifications supports the presence of within-batch temporal dependence, although the precise magnitude and decay time of the correlation remained model-dependent. Additional studies with multiple independently replicated composting batches in each season are required to determine whether the observed differences can be generalized as seasonal effects.

**Figure 3:**
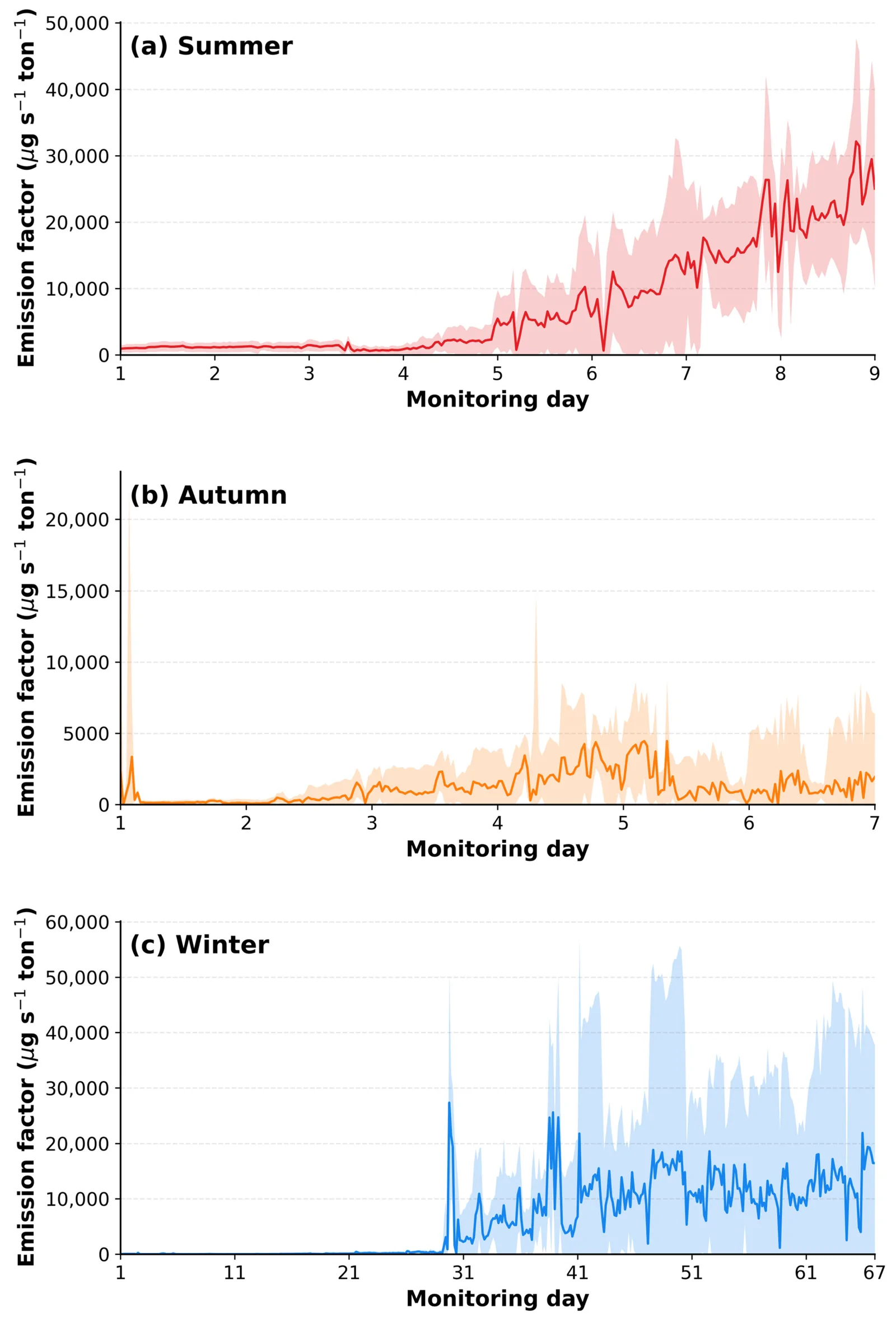
Temporal variation in CH_4_ emission factors during the monitoring periods: (**a**) summer, (**b**) autumn, and (**c**) winter. The shaded area represents the standard deviation within each measurement cycle.

**Figure 4:**
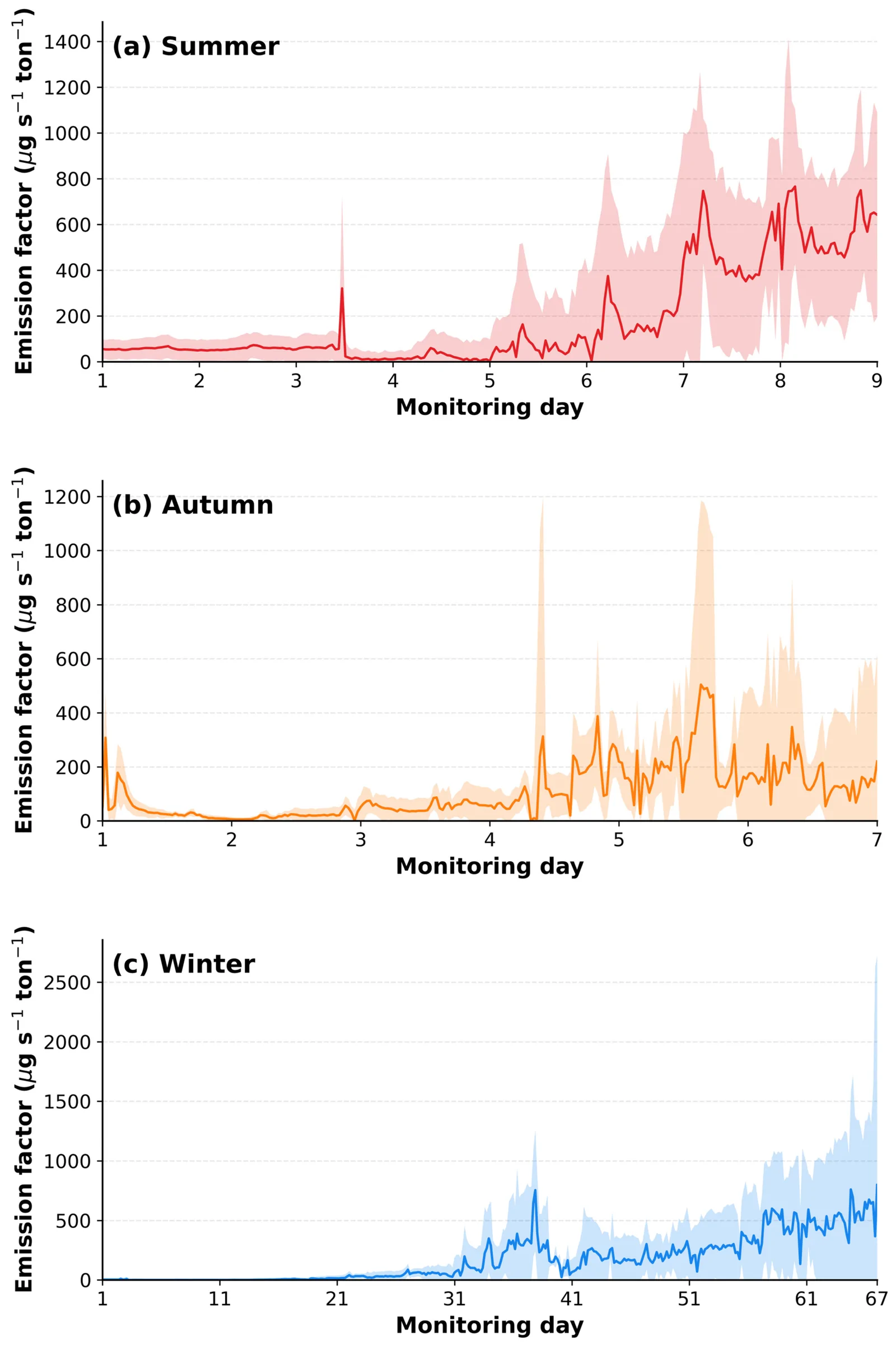
Temporal variation in N_2_O emission factors during the monitoring periods: (**a**) summer, (**b**) autumn, and (**c**) winter. The shaded area represents the standard deviation within each measurement cycle.

Figure 3 and Figure 4 show the seasonal patterns of greenhouse gas emission factors as composting progressed. Both methane and nitrous oxide emissions required some time to increase after the initial compost loading. This pattern aligns with previous findings showing that emissions rise several days after manure loading [40].

Ahn et al. [40] reported that composting 1200 kg of dairy manure for 80 days released 1.3 kg of methane and 0.0185 kg of nitrous oxide with turning. Without turning, the emissions were 0.9 kg of methane and 0.0053 kg of nitrous oxide. Husted [41] reported methane emissions of 34.57 gCH_4_·m^−3^·d^−1^ for cattle slurry and 42.7 gCH_4_·m^−3^·d^−1^ for solid cattle manure. Gonzalez-Avalos & Ruiz-Suarez [42] recorded an emission rate of 0.396 kgCH_4_·head^−1^·yr^−1^ when composting cattle manure collected from mid-latitude regions at 27 °C. Rahman et al. [43] observed emissions of 38 gCH_4_·head^−1^·d^−1^ and 26 gN_2_O·head^−1^·d^−1^ from feedlot pen surfaces. Leytem et al. [4] reported emissions of 0.49 kgCH_4_·head^−1^·d^−1^ and 0.01 kgN_2_O·head^−1^·d^−1^ from open dairy housing systems.

Applying Republic of Korea’s standard Hanwoo manure excretion rate of 7.98 kg·head^−1^·d^−1^ [44] to our results yields converted values of 1.28 kgCH_4_·head^−1^·yr^−1^ for methane and 0.039 kgN_2_O·head^−1^·yr^−1^ for nitrous oxide. Direct comparison with the previous literature is difficult due to differing experimental environments. However, considering that our study monitored a dedicated compost shed undergoing active composting, these values appear relatively low.

Also, the absence of internal compost pile temperature measurements is a major methodological limitation. Although changes in the C/N ratio and VS content provided information on changes in the compost material, they could not confirm the occurrence or duration of a thermophilic phase. Consequently, the thermal progression of composting and its relationship with CH_4_ and N_2_O emissions could not be evaluated.

The unique characteristics of our experimental setup may partly explain these lower values. The flux chamber in this study was modified from an actual farm compost shed, featuring a height of 4 m and a volume of 2400 m^3^. This setup is significantly larger than conventional flux chambers, which are typically measured in centimeters [45,46]. It is possible that this massive scale made it challenging to create a constant and uniform airflow inside the chamber.

This is also associated with the low ACH in our study. As the chamber volume increases, the ACH decreases, which may lead to an underestimation of gas emissions [47]. In addition, small-scale wind tunnel experiments demonstrate a directly proportional relationship between the ventilation rate and the mass transfer coefficient [48], and lower gas fluxes have been observed under low ACH conditions [49].

Workers entering the chamber for turning operations also disrupted the internal airflow patterns. Even during large-scale compost emissions research, small-scale flux chambers are typically preferred [49]. An uneven internal airflow might have prevented released greenhouse gases from immediately reaching the sensors. This limitation could have influenced the hourly analysis of emission factors.

Despite these various uncertainties, our study possesses a key strength in utilizing a full-scale operational composting facility. Instead of a small-scale experimental chamber, we utilized an actual operational compost shed under conventional farming practices. Disturbances from indoor-outdoor air exchange, farming operations, and compost turning could be seen as data artifacts. However, they allow us to evaluate comprehensive methane and greenhouse gas emission factors that incorporate selected real-world operational variables. In addition, monitoring across several seasons broadened the temporal coverage of the study.

### 3.3. Diurnal Patterns of Emission Factors

**Figure 5:**
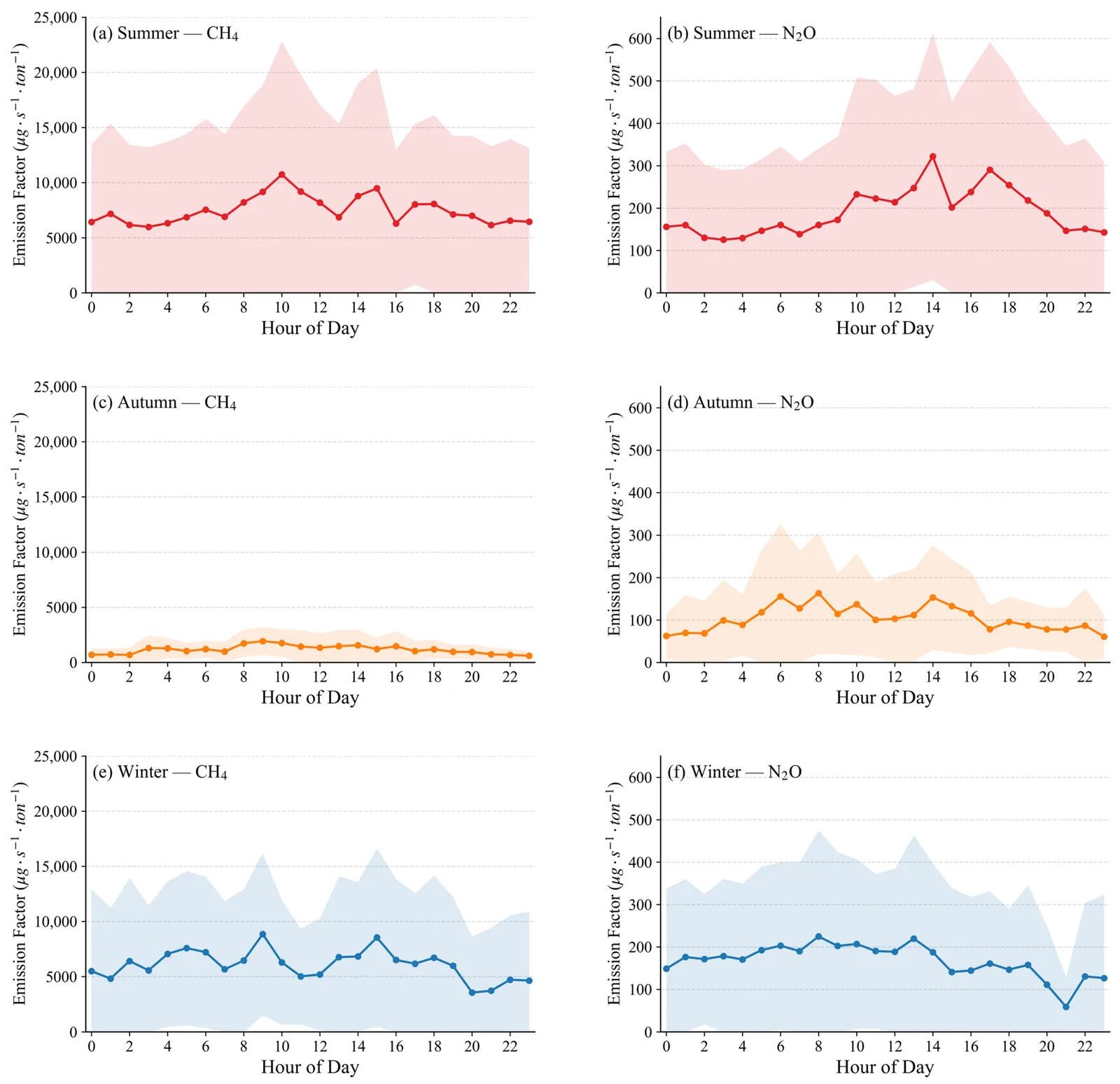
Mean temporal fluctuation in emission factors of greenhouse gases throughout the day; (**a**,**b**) summer, (**c**,**d**) autumn and (**e**,**f**) winter. Panels (**a**,**c**,**e**) show CH_4_ and (**b**,**d**,**f**) show N_2_O. Lines represent hourly means, and shaded areas indicate standard deviation for each hour of the day.

Figure 5 illustrates the seasonal variations in emission factors over a 24 h period. The data represent the hourly averages calculated from the 0 to 59 min marks of each hour. Regardless of the season, methane emissions peaked in the morning around 9:00 to 10:00 a.m. Because the inlet and outlet concentrations were measured sequentially rather than simultaneously, rapid changes occurring within a measurement cycle could affect the calculated concentration differences. The alternating inlet–outlet sequence reduced the temporal separation between the representative inlet and outlet measurements; however, short-lived emission changes, particularly during or immediately after compost turning, may have been temporally smoothed or shifted. Therefore, the diurnal patterns in Figure 5 should be interpreted as cycle-aggregated temporal trends rather than as measurements of the exact timing or magnitude of instantaneous emission peaks.

During summer and winter, methane emissions also showed a tendency to rise around 3:00 p.m. Nitrous oxide exhibited a similar trend, peaking around 9:00 a.m. before gradually declining. However, in summer, nitrous oxide emissions reached their highest level around 3:00 p.m.

These diurnal patterns may have been influenced by daily changes in ambient temperature and compost turning operations. During manure composting, emissions of greenhouse gases are strongly correlated with ambient temperature [50]. The higher hourly mean emissions observed around 3:00 p.m. coincided with the period of higher ambient temperature and may therefore partly reflect temperature-related changes in composting activity. However, the internal compost temperature was not monitored, and ambient air temperature may not accurately represent the thermal and microbial conditions within the compost pile. Moreover, no statistical analysis was performed to quantify the association between ambient temperature and the emission factors. Therefore, the observed afternoon pattern should be regarded as a plausible interpretation rather than evidence of a direct temperature effect.

Meanwhile, compost turning at the test farm was generally conducted during the morning. Turning operations during composting accelerate the release of gases from the pile [51,52]. It may have contributed to the higher hourly mean emissions observed around 9:00 a.m. However, emissions immediately before and after individual turning events were not compared statistically, and other time-dependent factors may also have contributed to the observed pattern. Accordingly, the morning emission pattern cannot be attributed exclusively to compost turning.

As shown in Table 3 and Figure 5, average gas emissions followed an unexpected seasonal order of summer, winter, and autumn. Throughout the total composting process, turning operations occurred 4 times in summer, 4 times in autumn, and 23 times in winter, corresponding to daily turning frequencies of 0.45 d^−1^ in summer, 0.57 d^−1^ in autumn, and 0.34 d^−1^ in winter. Although the winter batch had the greatest total number of turning events, it had the lowest daily turning frequency. The greater total number of winter turning events primarily reflected the longer composting duration and therefore cannot, by itself, explain the higher mean emission factors observed during winter. Turning may have caused transient increases in gas release, but its contribution to the mean emission factor of each batch was not quantified in this study.

## 4. Conclusions

This study measured CH_4_ and N_2_O emissions from three Hanwoo manure composting batches monitored during summer, autumn, and winter at a commercial cattle farm in Republic of Korea. Spring was not monitored directly, and autumn was used as a climatic proxy for spring. The mean emission factors followed a descending order of summer, winter, and autumn. The final estimated emission factors were 1.28 kgCH_4_·head^−1^·yr^−1^ and 0.039 kgN_2_O·head^−1^·yr^−1^, which were lower than values reported in several previous studies.

Exploratory rank-based comparisons identified nominal differences in emission-factor distributions among the three monitored composting batches. However, the strong temporal autocorrelation among repeated observations and the absence of independent batch replication within each season preclude interpreting these differences as definitive evidence of general seasonal effects.

The use of a full-scale composting facility allowed greenhouse gas emissions to be monitored under actual commercial operating conditions. However, the large chamber volume and non-uniform airflow may have prevented the sensors from capturing all gases released from the compost, potentially resulting in underestimation of the reported emission factors. Differences in facility design, operating conditions, and measurement methods should therefore be considered when comparing these values with those reported in previous studies.

The winter batch had a longer composting duration and a greater total number of turning events. Although these conditions may have increased cumulative emissions, they do not necessarily explain the higher mean emission factors observed during winter, and their individual effects were not quantified in this study.

The findings of this study are based on measurements from a single farm and are therefore insufficient for direct use as national emission factors. Nevertheless, they can serve as baseline data for future emission factor development. Further validation across multiple farms, manure management systems, and production scales is required to develop representative emission factors.

## Figures and Tables

**Table 1:** Moisture percentage, total Kjeldahl nitrogen (TKN), volatile solids (VS), C/N ratio and pH of manure. ‘Start’ and ‘End’ represent the analytical values at the initial and final stages of the composting process.

Factor	Season	Start	End
Moisture ^1^	Summer	61.2	42.0
	Autumn	58.7	45.9
	Winter	67.5	42.9
TKN ^2^	Summer	8526.9	4234.8
	Autumn	10,487.1	6252.2
	Winter	9853.0	4417.2
VS ^2^	Summer	291,640.7	144,637.6
	Autumn	296,516.9	141,855.3
	Winter	287,924.3	132,475.2
C/N ratio	Summer	23.6	19.8
	Autumn	23.1	19.4
	Winter	26.8	17.2
pH	Summer	8.61	8.54
	Autumn	8.74	8.06
	Winter	8.46	8.55

^1^ Unit: %. ^2^ Unit: mg·kg^−1^.

**Table 2:** Ambient temperature of the flux chamber during each monitoring period.

Monitoring Period	Minimum Temperature (°C)	Maximum Temperature (°C)	Mean Temperature (°C)
Summer	19.0	31.5	24.2
Autumn	7.5	22.4	13.6
Winter	−17.9	15.0	−1.5

**Table 3:** Descriptive statistics for greenhouse gas emission factors during each monitoring period.

Season	CH_4_			N_2_O		
	Mean	Median	SD	Mean	Median	SD
Summer	7502.89	2321.47	8095.58	185.85	61.48	214.97
Autumn	1174.36	929.48	1036.46	105.61	65.19	101.07
Winter	6141.51	4295.87	6484.01	169.00	100.19	186.88
Total *	5086.72	1432.97	6542.44	155.05	66.62	178.18

Unit: μg·s^−1^·ton^−1^. * Total represents descriptive statistics calculated from all retained observations pooled across the three monitored batches.

## Data Availability

The raw data supporting the conclusions of this article will be made available by the authors on request.

## References

[1] Schmithausen A.J., Trimborn M., Buescher W. (2018). Sources of nitrous oxide and other climate relevant gases on surface area in a dairy free stall barn with solid floor and outside slurry storage. Atmos. Environ..

[2] Sommer S.G., Clough T.J., Chadwick D., Petersen S.O. (2013). Greenhouse gas emissions from animal manures and technologies for their reduction. Anim. Manure Recycl. Treat. Manag..

[3] Borhan M.S., Capareda S.C., Mukhtar S., Faulkner W.B., McGee R., Parnell C.B. (2011). Greenhouse gas emissions from ground level area sources in dairy and cattle feedyard operations. Atmosphere.

[4] Leytem A.B., Dungan R.S., Bjorneberg D.L., Koehn A.C. (2011). Emissions of ammonia, methane, carbon dioxide, and nitrous oxide from dairy cattle housing and manure management systems. J. Environ. Qual..

[5] Owen J.J., Silver W.L. (2015). Greenhouse gas emissions from dairy manure management: A review of field-based studies. Glob. Change Biol..

[6] Phillips F.A., Wiedemann S., Naylor T.A., McGahan E., Warren B., Murphy C., Parkes S., Wilson J. (2016). Methane, nitrous oxide and ammonia emissions from pigs housed on litter and from stockpiling of spent litter. Anim. Prod. Sci..

[7] Beltran I., Van der Weerden T.J., Alfaro M.A., Amon B., de Klein C.A., Grace P., Hafner S., Hassouna M., Hutchings N., Krol D.J. (2021). DATAMAN: A global database of nitrous oxide and ammonia emission factors for excreta deposited by livestock and land-applied manure. J. Environ. Qual..

[8] Hassouna M., van der Weerden T.J., Beltran I., Amon B., Alfaro M.A., Anestis V., Cinar G., Dragoni F., Hutchings N.J., Leytem A. (2023). DATAMAN: A global database of methane, nitrous oxide, and ammonia emission factors for livestock housing and outdoor storage of manure. J. Environ. Qual..

[9] Liu Z., Liu Y., Murphy J.P., Maghirang R. (2017). Ammonia and methane emission factors from cattle operations expressed as losses of dietary nutrients or energy. Agriculture.

[10] Qu Q., Groot J.C., Zhang K., Schulte R.P. (2021). Effects of housing system, measurement methods and environmental factors on estimating ammonia and methane emission rates in dairy barns: A meta-analysis. Biosyst. Eng..

[11] Witkowska D., Korczyński M., Koziel J.A., Sowińska J., Chojnowski B. (2020). The effect of dairy cattle housing systems on the concentrations and emissions of gaseous mixtures in barns determined by Fourier-transform infrared spectroscopy. Ann. Anim. Sci..

[12] Vechi N.T., Mellqvist J., Scheutz C. (2022). Quantification of methane emissions from cattle farms, using the tracer gas dispersion method. Agric. Ecosyst. Environ..

[13] Ward N., Atkins A., Atkins P. (2024). Estimating methane emissions from manure: A suitable case for treatment?. Environ. Res. Food Syst..

[14] Du Toit C.J.L., Van Niekerk W. (2013). Direct methane and nitrous oxide emissions of South African dairy and beef cattle. S. Afr. J. Anim. Sci..

[15] Leitner S., Ring D., Wanyama G.N., Korir D., Pelster D.E., Goopy J.P., Butterbach-Bahl K., Merbold L. (2021). Effect of feeding practices and manure quality on CH4 and N2O emissions from uncovered cattle manure heaps in Kenya. Waste Manag..

[16] Chadwick D., Cardenas L., Dhanoa M., Donovan N., Misselbrook T., Williams J., Thorman R., McGeough K., Watson C.J., Bell M. (2018). The contribution of cattle urine and dung to nitrous oxide emissions: Quantification of country specific emission factors and implications for national inventories. Sci. Total Environ..

[17] Liu Z., Powers W., Liu H. (2013). Greenhouse gas emissions from swine operations: Evaluation of the Intergovernmental Panel on Climate Change approaches through meta-analysis. J. Anim. Sci..

[18] Wiedemann S., Phillips F.A., Naylor T.A., McGahan E., Keane O., Warren B., Murphy C. (2016). Nitrous oxide, ammonia and methane from Australian meat chicken houses measured under commercial operating conditions and with mitigation strategies applied. Anim. Prod. Sci..

[19] Ji E.S., Park K.-H. (2012). Methane and nitrous oxide emissions from livestock agriculture in 16 local administrative districts of Korea. Asian-Australas. J. Anim. Sci..

[20] Nugrahaeningtyas E., Jeong S.-H., Novianty E., Ataallahi M., Park G.W., Park K.-H. (2023). Measurement of greenhouse gas emissions from a dairy cattle barn in Korea. J. Anim. Sci. Technol..

[21] Paik C., Chung Y., Kim H., Kim Y.J. (2016). Comparison of emission estimates for non-CO2 greenhouse gases from livestock and poultry in Korea from 1990 to 2010. Anim. Sci. J..

[22] Park Y., Yeon S., Park K.-H. (2020). Development of GHG emission factors for the life cycle of the animal manure treatment systems. J. Korean Soc. Environ. Eng..

[23] Won S., Cho W., Lee J., Park K., Ra C. (2014). Data build-up for the construction of Korean specific greenhouse gas emission inventory in livestock categories. Asian-Australas. J. Anim. Sci..

[24] Statistics Korea (2023). Amount of Production by Agricultural and Forestry Sector.

[25] Ministry of Climate, Energy and Utilities (2025). Livestock Manure Generation and Treatment Status.

[26] Won S., Yoon Y., Hamid M.M.A., Reza A., Shim S., Kim S., Ra C., Novianty E., Park K.-H. (2020). Estimation of greenhouse gas emission from Hanwoo (Korean native cattle) manure management systems. Atmosphere.

[27] Kang K., Cho H., Lee M., Jeong S., Jeon S., Kang K., Kang H., Shin J., Ahn H., Seo S. (2026). A multi-chamber incubation system for accurate assessment of ammonia and hydrogen sulfide emissions from cattle manure: Effects of dietary protein level. Anim. Feed. Sci. Technol..

[28] Bai M., Sun J., Dassanayake K.B., Benvenutti M.A., Hill J., Denmead O.T., Flesch T., Chen D. (2016). Non-interference measurement of CH_4_, N_2_O and NH_3_ emissions from cattle. Anim. Prod. Sci..

[29] Lee W.-J., Park G.-W., Kim K.-Y., Kim J.-K. (2026). Seasonal Variation and Operational Impacts on Ammonia Emission Factors from Hanwoo Manure Composting. Atmosphere.

[30] Wheeler E.F., Topper P., Brandt R., Brown N., Adviento-Borbe A., Thomas R., Varga G. (2011). Multiple-chamber instrumentation development for comparing gas fluxes from biological materials. Appl. Eng. Agric..

[31] Perez R.R.A. (2011). Evaluation and Application of a Dynamic Emissions Chamber for Quantifying Gaseous Emissions from Laying Hen Manure.

[32] Kim K.Y., Ko H.J., Kim H.T. (2012). Comparison of seasonal concentration of ammonia and hydrogen sulfide in swine house according to pig’s growth stage. J. Agric. Life Sci..

[33] Oh S., Kim S.-G., Lee J.B., Park J., Jee J.-B., Hong S.-W., Kwon K.-S., Song M. (2021). Spatial distributions of atmospheric ammonia in a rural area in South Korea and the associated impact on a nearby urban area. Atmosphere.

[34] Administration K.M. Seasonal length. Climate Information Portal. https://www.climate.go.kr/home/CCS/contents_2021/influence/inf_2-1.php.

[35] Ji Z., Zhang L., Liu Y., Li X., Li Z. (2023). Evaluation of composting parameters, technologies and maturity indexes for aerobic manure composting: A meta-analysis. Sci. Total Environ..

[36] Shen B., Zheng L., Zheng X., Yang Y., Xiao D., Wang Y., Sheng Z., Ai B. (2024). Insights from meta-analysis on carbon to nitrogen ratios in aerobic composting of agricultural residues. Bioresour. Technol..

[37] Sullivan D.M., Miller R.O. (2001). Compost quality attributes, measurements and variability. Compost Utilization in Horticultural Cropping Systems.

[38] Siegel S. (1988). The Kruskal-Wallis one-way analysis of variance by rank. Handbook of Parametric and Nonparametric Statistical Procedures.

[39] Divine G.W., Norton H.J., Barón A.E., Juarez-Colunga E. (2018). The Wilcoxon–Mann–Whitney procedure fails as a test of medians. Am. Stat..

[40] Ahn H.K., Mulbry W., White J., Kondrad S. (2011). Pile mixing increases greenhouse gas emissions during composting of dairy manure. Bioresour. Technol..

[41] Husted S. (1994). Seasonal Variation in Methane Emission from Stored Slurry and Solid Manures.

[42] Gonzalez-Avalos E., Ruiz-Suarez L. (2001). Methane emission factors from cattle manure in Mexico. Bioresour. Technol..

[43] Rahman S., Borhan M.S., Swanson K. (2013). Greenhouse gas emissions from beef cattle pen surfaces in North Dakota. Environ. Technol..

[44] Rural Development Administration (2025). Livestock Excreta Emission Source Unit.

[45] Parker D.B., Casey K.D., Todd R.W., Waldrip H.M., Marek G.M., Auvermann B.W., Marek T.H., Webb K., Willis W.M., Pemberton B. (2017). Improved chamber systems for rapid, real-time nitrous oxide emissions from manure and soil. Trans. ASABE.

[46] Cattaneo M., Tayà C., Burgos L., Morey L., Noguerol J., Provolo G., Cerrillo M., Bonmatí A. (2023). Assessing ammonia and greenhouse gas emissions from livestock manure storage: Comparison of measurements with dynamic and static chambers. Sustainability.

[47] Eckley C., Gustin M., Lin C.-J., Li X., Miller M. (2010). The influence of dynamic chamber design and operating parameters on calculated surface-to-air mercury fluxes. Atmos. Environ..

[48] Ye Z., Zhang G., Li B., Strøm J.S., Tong G., Dahl P.J. (2008). Influence of airflow and liquid properties on the mass transfer coefficient of ammonia in aqueous solutions. Biosyst. Eng..

[49] Pedersen J., Hafner S.D., Pacholski A., Karlsson V.I., Rong L., Labouriau R., Kamp J.N. (2024). Evaluation of optimized flux chamber design for measurement of ammonia emission after field application of slurry with full-scale farm machinery. Atmos. Meas. Tech..

[50] Zhu X., Dong W., Wang H., Yan C., Liu H., Liu E. (2017). Effects of cattle manure composting methods on greenhouse gas and ammonia emissions. Trans. Chin. Soc. Agric. Eng..

[51] Abd El Kader N., Robin P., Paillat J.-M., Leterme P. (2007). Turning, compacting and the addition of water as factors affecting gaseous emissions in farm manure composting. Bioresour. Technol..

[52] Shao H., Yi S., Shen Y., Meng H., Ding J., Cheng H., Zhou H., Zhang D. (2024). Effects of different processes on gas emission during composting of cow manure. Trans. Chin. Soc. Agric. Eng..

